# Validity of current surgical strategies for aneurysmal progression in post-subacute Stanford type B aortic dissection: a propensity score–matched comparative analysis

**DOI:** 10.3389/fcvm.2025.1696381

**Published:** 2026-01-09

**Authors:** Ken Nakamura, Kentaro Akabane, Shusuke Arai, Ryota Katsura, Miku Konaka, Jun Hayashi, Cholsu Kim, Hideaki Uchino, Takao Shimanuki, Tetsuro Uchida

**Affiliations:** 1 Division of Cardiovascular Surgery, Nihonkai General Hospital, Sakata, Japan; 2 Second Department of Surgery, Yamagata University Faculty of Medicine, Yamagata, Japan

**Keywords:** aortic aneurysm, best medical therapy, graft replacement, preemptive thoracic endovascular aortic repair, uncomplicated Stanford type b aortic dissection

## Abstract

**Objective:**

The use of preemptive thoracic endovascular aortic repair (TEVAR) has been expanding, especially in cases where aortic enlargement is an indication for surgical intervention. Current guidelines recommend treatment for chronic type B aortic dissection (TBAD) when aortic diameter increases by ≥5 mm over a 6-month period. However, the optimal window for preemptive TEVAR is the subacute phase (up to 3 months), creating a dilemma where intervention may be delayed beyond this window. This study investigates the short- and long-term outcomes of aggressive treatment for rapidly enlarging aneurysms.

**Subjects and methods:**

Between July 2004 and August 2024, 432 patients with acute Stanford type B aortic dissection were treated at two centers. Of these, 324 patients who completed acute best medical therapy (BMT) were included. Patients with rapid enlargement of aortic aneurysms who did not meet absolute surgical indications (aneurysm diameter <55 mm and <5 mm/month) were compared to those who continued BMT. Propensity score matching was performed between the BMT group (Group B, *n* = 83) and the aortic expansion group (Group E, *n* = 83).

**Results:**

Among the 324 eligible patients, 88 exhibited rapid aortic enlargement (≤5 mm in <6 months). Of these, 42 (48%) underwent surgical intervention: 12 (14%) received graft replacement, and 30 (34%) underwent TEVAR, with 28 (32%) receiving preemptive TEVAR. The BMT group continued without surgery, with 7 patients (3%) receiving graft replacement and 20 (8%) undergoing TEVAR. In Group E, aortic diameter significantly increased compared to Group B (0.4 ± 0.2 vs. 1.1 ± 0.2 mm, *p* < 0.001). The false lumen was thrombosed in all preemptive TEVAR patients (mean 3.2 ± 5.1 months). In addition, univariate Cox regression identified preemptive TEVAR as a significant protective factor against aorta-related events (HR: 0.03, 95% CI: 0.004–0.22, *p* < 0.001), highlighting its potential role in reducing adverse aortic outcomes. The aorta-related death-free survival rates at 1, 5, and 10 years were 99/99/99% vs. 99/93/88% for Groups B and E, respectively (*p* = 0.097). Seven deaths occurred in Group E, with six related to ruptured aortic aneurysms.

**Conclusion:**

Selective preemptive TEVAR for patients with rapid aortic enlargement in the subacute and chronic phases of TBAD showed favorable outcomes. Although no significant difference in aorta-related mortality was found, the higher incidence of rupture in untreated patients suggests that preemptive TEVAR may offer a benefit. Further research is needed to identify the patients most likely to benefit from early surgical intervention.

## Introduction

Acute aortic dissection is reported to occur with a frequency of 3.6%–10% per 100,000 population in Japan (1, 2). Given the high mortality associated with the condition, efforts are underway to explore improved treatment strategies. Thoracic endovascular aortic repair (TEVAR) for uncomplicated type B aortic dissection (TBAD) has recently been reported to improve long-term prognosis (3, 4), prompting ongoing discussions regarding optimal patient selection and timing of intervention (5, 6). Current treatment guidelines recommend intervention for chronic TBAD when the descending thoracic aorta reaches a diameter of 55–60 mm. For type B residual dissection, treatment is indicated when there is an aortic enlargement of 5 mm or more over a 6-month period (1, 7). However, the optimal window for preemptive TEVAR is considered to be the subacute phase (within 3 months) (8–10), creating a clinical dilemma: by the time aortic enlargement of ≥5 mm is confirmed after 6 months, the ideal timing for intervention may already have passed. Our group has proactively performed preemptive TEVAR in cases of uncomplicated TBAD showing rapid aortic expansion over a short period, even in the absence of formal surgical indications. However, the prognostic benefit of selective invasive intervention in high-risk TBAD patients with rapid aortic growth remains unestablished. Therefore, we conducted a retrospective study to investigate this issue.

## Subjects and methods

The institutional ethical review board approved the research protocol and patient written consent were waived because of the retrospective nature of the study (Institutional Review Board #007-3-9). All patients were informed about the use of their data for clinical research.

**Figure 1:**
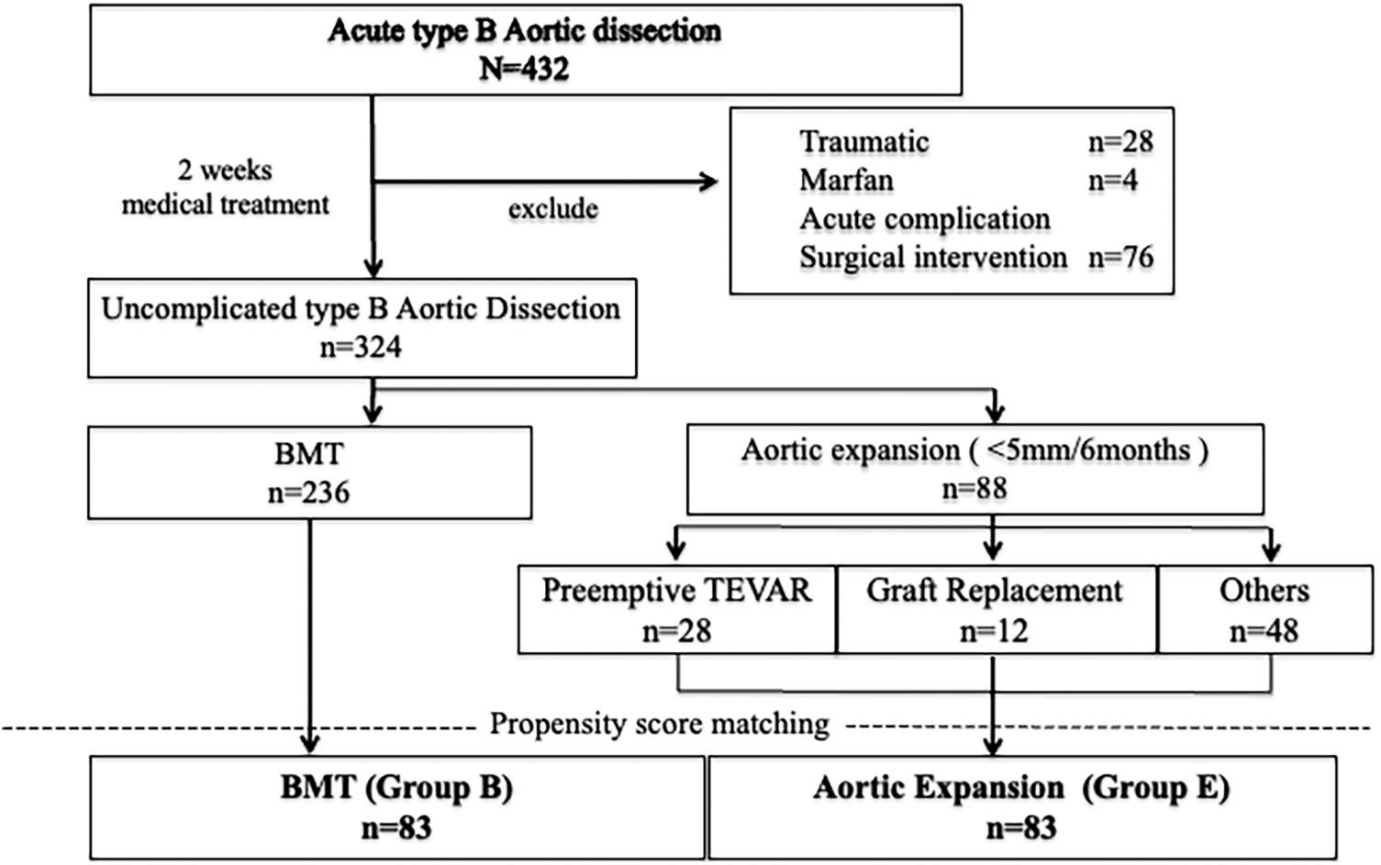
Flow diagram of all patients with Stanford type B aortic dissection included in this study. A total of 432 patients with acute type B aortic dissection were initially enrolled. Among them, 324 patients without complications were included for analysis and classified into two groups: the best medical therapy (BMT) group (*n* = 236) and the aortic expansion group (*n* = 88). The aortic expansion group consisted of patients who underwent preemptive thoracic endovascular aortic repair (TEVAR; *n* = 28), graft replacement (*n* = 12), or were managed conservatively (*n* = 48). Propensity score matching was performed between the BMT group and the aortic expansion group to adjust for baseline characteristics. After matching, 83 patients were included in each group [BMT group (Group B) and aortic expansion group (Group E)] for comparative analysis.

Two centers in Yamagata prefecture participated in this study. Data were collected between July 2004 and August 2024 in Yamagata University Hospital and between February 2016 and January 2025 in Nihonkai General Hospital (Figure 1). All patients at both centers were managed according to the same diagnostic criteria and Japanese guideline–based protocols, including standardized blood pressure control and imaging schedules. Any minor variations in practice were harmonized through regular inter-center meetings and consensus discussions to ensure uniform management. Acute onset of aortic dissection was defined as a case in which the patient was examined and diagnosed with pain and other symptoms as the primary complaint. Acute uncomplicated TBAD was defined as the absence of pain or other symptoms, malperfusion (both dynamic obstruction, which is improved by false lumen decompression, and static obstruction, which is not improved) or signs of early disease progression such as type A dissection presenting within 14 days of symptom onset (1, 7). As outlined in the guidelines, phases were defined as acute (<14 days), subacute (15–90 days), and chronic (>90 days) from symptom onset (1, 7). The term “early chronic” denotes the period immediately following day 90. On admission, patients were treated in the intensive care unit (ICU) or a high care unit (HCU). All patients underwent contrast-enhanced computed tomography (CT) scanning at emergency admission, and on the 1st and 7th days following admission. Patients were eligible for discharge 4 weeks after onset. This protocol was developed based on the Japanese guidelines (1). During follow-up, patients who had an aorta-related event, despite medical management, underwent an aortic intervention. The term “aorta-related event” refers to any of the following: enlargement of the aortic diameter (≥55 mm or ≥5 mm enlargement within 6 months), malperfusion, or aortic rupture (1, 7). In this study, an aortic diameter enlargement of >5 mm within 6 months was defined as a secondary indicator of aortic instability, clearly distinguished from the absolute surgical indication of aneurysmal dilation ≥55 mm. TEVAR performed after 6 months was classified as a therapeutic intervention in the chronic phase rather than as preemptive treatment. Chronic-phase enlargement events were documented as follow-up endpoints and were not used as baseline exclusion criteria. CT angiography (CTA) images obtained at presentation, as well as follow-up CT scans, were reviewed for all patients. The standard scan regimen was as follows: at symptom onset, at discharge, 3 or 6 months after discharge, 12 months after discharge and yearly thereafter. The scan regimen differed for individual patients, depending on findings. All imaging studies were reviewed for radiologic signs of adverse events, such as organ malperfusion or rupture, as well as imaging evidence of pathology resolution. These measures follow the same methodology as in our previous report (9, 11, 12). Preemptive TEVAR was defined as TEVAR prior to the occurrence of an aortic adverse event, as defined in the guideline and in previous reports (1, 7, 9). The indication for preemptive TEVAR was determined comprehensively based on individual patient risk factors, including an aortic diameter ≥40 mm, a narrow true lumen, a large or enlarging false lumen, a patent false lumen, and a large primary entry (≥10 mm), together with the patient's informed and proactive request after sufficient explanation. Interventions performed in response to the occurrence of an aortic adverse event were classified separately as event-driven TEVAR. Stent-graft placement was performed using four commercially available devices: Cook (*n* = 19, 67%), Medtronic (*n* = 3, 11%), GORE (*n* = 3, 11%), and TERUMO (*n* = 3, 11%). Device diameter was selected to match the nondissected proximal aortic diameter, in accordance with each manufacturer's instructions for use, and all devices were deployed in the true lumen. The proximal landing zone was measured during systole, ensuring placement in at least 2 cm of healthy, nondissected aorta. In Zone 2 cases, revascularization was performed when necessary to maintain adequate perfusion of the left subclavian artery, including axillo–axillary bypass as appropriate. When sufficient antegrade perfusion was preserved, Zone 2 repair was performed without debranching using simple proximal coverage. A total of 324 CT images and their analysis were available for routine surveillance. Image analysis was performed on a SYNAPSE VINCENT system (Tokyo Japan, FUJIFILM Holding Corporation) with a dedicated 3D image analyzer. All CTA measurements were obtained using multiplanar reconstruction (MPR) images in an axial plane perpendicular to the aortic median centerline. The aortic median centerline was generated by software on the VINCENT system that uses a 3D algorithm to perform MPR centered on the contrast-enhanced aortic lumen. In no instance did the maximum aortic diameter occur in a non-dissected segment. The diameters of the true and false lumen were measured. Measurements were performed on the orthogonal cross-section in which the aortic diameter was maximal. Aortic growth rate was calculated by dividing the change in aortic diameter by the time interval (six months or one year) from symptom onset. The status of the false lumen on imaging was classified as “patent” if there was flow without thrombus, and “partial thrombosis” was assessed in the later phase of contrast CT.

Clinical characteristics and CT imaging parameters of patients at emergency admission and during follow-up were summarized. Categorical measures were presented as percentages. Continuous measures were presented as medians and interquartile ranges. Outcomes were assessed with unadjusted and multivariable models. Aorta-related event-free rates were calculated using Kaplan–Meier curves and examined by the log-rank test, with aorta-related events defined as enlargement of the aortic diameter (≥55 mm or ≥5 mm enlargement within 6 months), malperfusion, or aortic rupture, and aorta-related mortality defined as death due to aortic rupture. All model-based results are presented with 95% confidence intervals. This study was performed under intention-to-treat analysis. A matched group analysis was performed by propensity matching preemptive TEVAR cases and control (CTR) cases. Propensity scores were generated using logistic regression analyses in 2 steps. Potential predictors were selected from a published data review, known confounding covariates for the outcomes of interest, differences between the 2 patients groups (Table 1), and clinical judgement. The groups were matched with age, sex, body mass index, history of chronic obstructive pulmonary disease, hypertension, diabetes mellitus and hyperlipidemia. Univariate analysis was performed using ANOVA or Student's *t*-test and the Chi-square test. In addition, univariable Cox proportional hazards regression analysis was conducted using the entire study cohort to evaluate clinical predictors of aorta-related events. Hazard ratios with 95% confidence intervals were calculated for each variable included in the analysis. All statistical analyses were performed with JMP software, ver. 18 (SAS Institute, Tokyo, Japan).

**Table 1:** Baseline characteristics and follow-up computed tomography findings of patients with and without rapid aortic enlargement.

Characteristics	All patients (*n* = 324)	After propensity score matching		
		BMT (Group B) (*n* = 83)	Aortic enlargement (Group E) (*n* = 83)	*P*-values
Age, y, Mean ± SD	70.1 ± 12.1	69.5 ± 11.9	70.3 ± 10.4	0.63
Male, %	71 (230 of 324)	75 (62 of 83)	73 (60 of 83)	0.86
Height, cm, Mean ± SD	161.2 ± 9.5	162.5 ± 8.7	160.6 ± 9.0	0.18
Weight, kg, Mean ± SD	61.9 ± 14.4	63.3 ± 13.2	60.8 ± 12.5	0.21
BMI (kg/m^2^), Mean ± SD	23.7 ± 4.3	23.7 ± 3.7	23.5 ± 3.7	0.64
COPD, %	40 (128 of 324)	36 (30 of 83)	40 (33 of 83)	0.75
HTN, %	98 (317 of 324)	99 (82 of 83)	98 (81 of 83)	1.00
Diabetes Mellitus, %	8.0 (26 of 324)	4.8 (4 of 83)	6.0 (5 of 83)	0.51
History of stroke, %	7.1 (23 of 324)	6.0 (5 of 83)	4.8 (4 of 83)	1.00
Renal insufficiency, %	13.0 (42 of 324)	14.5 (12 of 83)	10.8 (9 of 83)	0.64
Coronary artery disease, %	4.3 (14 of 324)	2.4 (2 of 83)	3.6 (3 of 83)	1.00
Hyperlipidemia, %	32 (103 of 324)	37 (31 of 83)	34 (28 of 83)	0.75
Follow up period, months, Mean ± SD	75 ± 54	77 ± 56	78 ± 59	0.93
Acute course of disease				
Delirium, %	46 (145 of 317)	43.4 (36 of 83)	46.9 (38 of 81)	0.75
Delirium onset, day %	2.4 (1.0–3.0)	2.2 (0.25–3.0)	2.8 (1.0–4.0)	0.68
NPPV required, %	26 (82 of 317)	25 (21 of 83)	25 (20 of 81)	1.00
Tracheal Intubation, %	8 (25 of 317)	14 (12 of 83)	7 (6 of 81)	0.21
Positive pressure ventilation required (NPPV or mechanical ventilation), %	29 (91 of 316)	37 (31 of 83)	28 (22 of 80)	0.19
ICU + HCU stay, day Mean ± SD	6.2 ± 4.5	7.0 ± 5.1	6.2 ± 4.8	0.32
Medication at discharge from the hospital				
Beta blockers n, %	80 (245 of 307)	80 (64 of 80)	72 (252 of 72)	0.34
Angiotensin converting enzyme inhibitors, %	18 (54 of 307)	20 (16 of 80)	25 (18 of 72)	0.56
Angiotensin II receptor blockers,%	61 (188 of 306)	63 (50 of 80)	51 (37 of 72)	0.19
Calcium channel blockers,%	79 (243 of 307)	83 (66 of 80)	74 (53 of 72)	0.24
Alpha blockers, %	29 (89 of 307)	36 (29 of 80)	25 (18 of 72)	0.16
Thiazide	4 (13 of 307)	4 (3 of 80)	6 (4 of 72)	0.71
Loop diuretics, %	5 (15 of 307)	4 (3 of 80)	7 (5 of 72)	0.48
Aldosterone antagonist, %	7 (22 of 307)	13 (10 of 80)	4 (3 of 72)	0.08
Statins,%	38 (117 of 307)	45 (36 of 80)	33 (24 of 72)	0.18
Steroids,%	4 (11 of 311)	2 (2 of 81)	8 (6 of 75)	0.15
Anticoagulants,%	11 (35 of 312)	14 (11 of 81)	13 (10 of 75)	1.00
Image findings				
Aortic diameter in early subacute, mm, Mean ± SD	38 ± 6	38 ± 5	39 ± 7	0.21
True lumen aortic diameter (TLD) (mm)	25 ± 7	25 ± 7	25 ± 9	0.98
False lumen aortic diameter (FLD) (mm)	12 ± 6	12 ± 6	12 ± 7	0.78
Rate of true/false lumen diameter (TLD/FLD)	3.2 ± 3.8	3.2 ± 3.9	3.4 ± 3.6	0.82
Entry located distal arch, %	80 (248 of 311)	89 (70 of 79)	72 (57 of 79)	0.02
Entry located tracheal bifurcation level, %	5 (16 of 311)	1 (1 of 79)	8 (6 of 79)	0.12
Entry located diaphragm level, %	9 (28 of 311)	9 (7 of 79)	10 (8 of 79)	1.00
Entry located celiac artery level, %	4 (12 of 311)	1 (1 of 79)	8 (6 of 79)	0.12
Entry located abdominal aorta, %	3 (9 of 311)	1 (1 of 79)	4 (3 of 79)	0.62
Number of intimal tears	2.0 ± 1.3	2.0 ± 1.2	2.2 ± 1.6	0.50
Diameter of primary entry, mm, Mean ± SD	10.8 ± 6.6	10.5 ± 5.9	12.7 ± 5.8	0.07
Patent false lumen, %	41 (133 of 324)	58 (48 of 83)	61 (51 of 83)	0.75
Perfused with partial thrombosis, %	19 (60 of 324)	22 (18 of 83)	13 (11 of 83)	0.38
Follow-up findings				
Aortic growth rate (mm/month)	0.50 ± 1.33	0.4 ± 0.2	1.1 ± 0.2	<0.001
Thorombosis of the false lumen, after preemptive TEVAR, %	100 (47 of 47)	100 (7 of 7)	100 (28 of 28)	—

TEVAR, thoracic endovascular aortic repair; BMT, best medical therapy; SD, standard deviation; BMI, body mass index; COPD, chronic obstructive pulmonary disease; HTN, hypertension; NPPV, noninvasive positive pressure ventilation; ICU, intensive care unit; HCU, high care unit.

## Results

A total of 432 patients were admitted with a diagnosis of acute TBAD. Of these, 324 patients (Center 1: *n* = 176; Center 2: *n* = 148) who received medical management during the acute phase and continued best medical therapy (BMT) into the subacute phase were included in the study. Follow-up computed tomography (CT) beyond the subacute phase was not performed in 20 patients; of these, 10 underwent non-contrast (plain) CT only. All patients were Japanese, and 230 (71%) were male. The mean age was 70.1 years (range, 34–96). The most common comorbidity was hypertension (*n* = 317, 98%), followed by diabetes mellitus (*n* = 26, 8.0%) and renal insufficiency (*n* = 42, 13.0%). The mean follow-up period was 75 ± 54 months (median: 69 months, interquartile range: 28.25–109.5 months) (Table 1, Supplementary Table 1). During follow-up, 67 patients (20.7%) required surgical intervention due to aortic adverse events, among whom 47 (14.5%) underwent preemptive TEVAR. Of these, 29 procedures were performed in Zone 2, 7 in Zone 3, and 11 in other zones. All preemptive TEVARs were indicated based on rapid aortic enlargement, although none of the patients met the conventional criterion of a maximum aortic diameter exceeding 55 mm, as the expansion occurred over a short interval. No cases of stent graft placement proximal to Zone 2 were performed as part of preemptive TEVAR. Eleven patients (3.4%) died from aortic adverse events during the follow-up period; notably, there were no aorta-related deaths among the 47 patients who underwent preemptive TEVAR. In 88 patients, the aortic diameter increased rapidly—though by less than 5 mm—within 6 months. Among these, 42 patients (48%) underwent surgical intervention: 12 (14%) received open graft replacement and 30 (34%) underwent TEVAR, including 28 patients (32%) who received preemptive TEVAR. Best medical therapy (BMT) was continued in the remaining 236 patients, of whom 7 (3%) underwent graft replacement and 20 (8%) underwent TEVAR during follow-up. Propensity score matching was performed to compare outcomes between the BMT group (Group B, *n* = 83) and the rapid aortic expansion group (Group E, *n* = 83)(Figure 1), significant differences were obtained in aortic growth rate (0.4 ± 0.2 vs. 1.1 ± 0.2, *p* < 0.001), entry located distal arch (89%,70 of 79 vs. 72%, 57 of 79, *p* = 0.02), and diameter of primary entry (10.5 ± 5.9 vs. 12.7 ± 5.8, *p* = 0.07) (Table 1, Supplementary Table 2).

Among all therapeutic interventions, 70% were performed in the subacute phase and 30% in the early chronic phase. Preemptive TEVAR, however, was performed much earlier, with a mean time-to-procedure of 1.4 months (42 days) and a median of 1 month (30 days), and all procedures were conducted within the subacute phase (≤90 days). The false lumen was thrombosed in all patients (mean: 3.2 ± 5.1 months) after the procedure. At 12 months after preemptive TEVAR, the aortic diameter at the site of maximal dissection was smaller in Group B than in Group E (37.6 ± 8.0 mm vs. 41.4 ± 8.6 mm, *p* = 0.009), showing a trend toward expansion in Group E. The maximum aortic diameter during the observation period was also significantly larger in Group E (38.6 ± 6.3 mm vs. 44.7 ± 8.5 mm, *p* < 0.0001), accompanied by a greater false lumen diameter (10.9 ± 10.3 mm vs. 14.6 ± 10.7 mm, *p* < 0.05) (Table 2). In addition, univariate Cox regression demonstrated that preemptive TEVAR was significantly associated with a lower risk of aorta-related events (HR: 0.03, 95% CI: 0.004–0.22, *p* < 0.001), indicating its potential protective effect against adverse aortic outcomes (Table 3). Among patients in Group E who underwent preemptive TEVAR, the postoperative course was generally favorable without the need for early reintervention; however, in two cases (7.1%), residual false lumen perfusion was associated with progression of distal aortic dissection, potentially requiring additional intervention in the future.

**Table 2:** Longitudinal aortic diameter measurements before and after intervention in groups B and E after propensity score matching.

Longitudinal CT assessment before and after intervention	Measured Lumen	After propensity score matching aortic diameter, mm, Mean ± SD		
		BMT (Group B) (*n* = 83)	Aortic enlargement (Group E) (*n* = 83)	*P*-values
Early subacute	True + False lumen	37.9 ± 4.8	39.0 ± 6.5	0.21
	True lumen	25.4 ± 7.0	25.4 ± 9.2	0.98
	False lumen	12.1 ± 6.1	12.4 ± 6.6	0.78
Immediately after preemptive TEVAR or follow-up CT in the subacute phase	True + False lumen	37.7 ± 6.8	39.7 ± 6.0	0.07
	True lumen	25.8 ± 7.2	27.0 ± 9.4	0.40
	False lumen	12.4 ± 6.7	13.0 ± 8.1	0.65
12-month after TEVAR or follow-up CT approximately 12-month after onset	True + False lumen	37.6 ± 8.0	41.4 ± 8.6	0.01
	True lumen	28.8 ± 7.6	31.3 ± 9.1	0.08
	False lumen	9.3 ± 9.8	10.2 ± 10.6	0.64
Max aortic diameter	True + False lumen	38.6 ± 6.3	44.7 ± 8.5	<0.001
	True lumen	27.7 ± 8.1	28.7 ± 11.3	0.54
	False lumen	10.9 ± 10.3	14.6 ± 10.7	0.04

Aortic diameters of the true lumen, false lumen, and combined true + false lumen were measured at defined time points throughout the disease course. “Immediately after preemptive TEVAR or follow-up CT in the subacute phase” represents the first available post–subacute-phase imaging for each patient, applicable to both groups. “12-month after TEVAR or follow-up CT approximately 12-month after onset” indicates imaging obtained around 12-month after symptom onset, regardless of whether TEVAR was performed.

SD, standard deviation; TEVAR, thoracic endovascular aortic repair; BMT, best medical therapy.

**Table 3:** Univariable Cox proportional hazards regression analysis performed in the entire study cohort to identify clinical predictors of aorta-related events. Hazard ratios (HRs) with 95% confidence intervals (CIs) are presented for each variable.

Variables	Hazard ratio	95% confidence interval	*P*-values
Age	4.41	(2.08, 9.41)	<0.001
Male	0.90	(0.68, 1.21)	0.49
BMI	0.51	(0.22, 1.15)	0.10
HT	0.45	(0.16, 1.21)	0.16
Diabetes Mellitus	1.37	(0.83, 2.26)	0.23
History of stroke	3.18	(1.91, 5.30)	<0.001
Renal insufficiency	1.53	(1.04, 2.24)	0.03
Coronary artery disease	1.59	(0.80, 3.15)	0.21
Hyperlipidemia	0.81	(0.61, 1.08)	0.15
COPD	1.46	(1.10, 1.93)	0.01
Delirium	1.37	(1.05, 1.80)	0.02
ICU + HCU stay	1.01	(0.99, 1.04)	0.29
Beta blockers	1.09	(0.77, 1.56)	0.59
Steroid	0.69	(0.28, 1.70)	0.39
Anticoagulant	2.10	(1.35, 3.28)	<0.01
Aortic diameter in early subacute	1.02	(0.99, 1.04)	0.07
Maximum Aortic Diameter >40 mm	0.56	(0.42, 0.76)	<0.001
Number of intimal tears	0.78	(0.65, 0.92)	<0.01
Diameter of primary entry	0.93	(0.90, 0.97)	<0.001
Patent false lumen	1.45	(1.08, 1.93)	0.01
Preemptive TEVAR	0.01	(0.01, 0.22)	<0.001
Entry located distal arch	1.69	(1.16, 2.48)	<0.01

HR, hazard ratio; CI, confidence interval; BMI, body mass index; HT, hypertension; COPD, chronic obstructive pulmonary disease; ICU, intensive care unit; HCU, high care unit; TEVAR, thoracic endovascular aortic repair.

**Figure 2:**
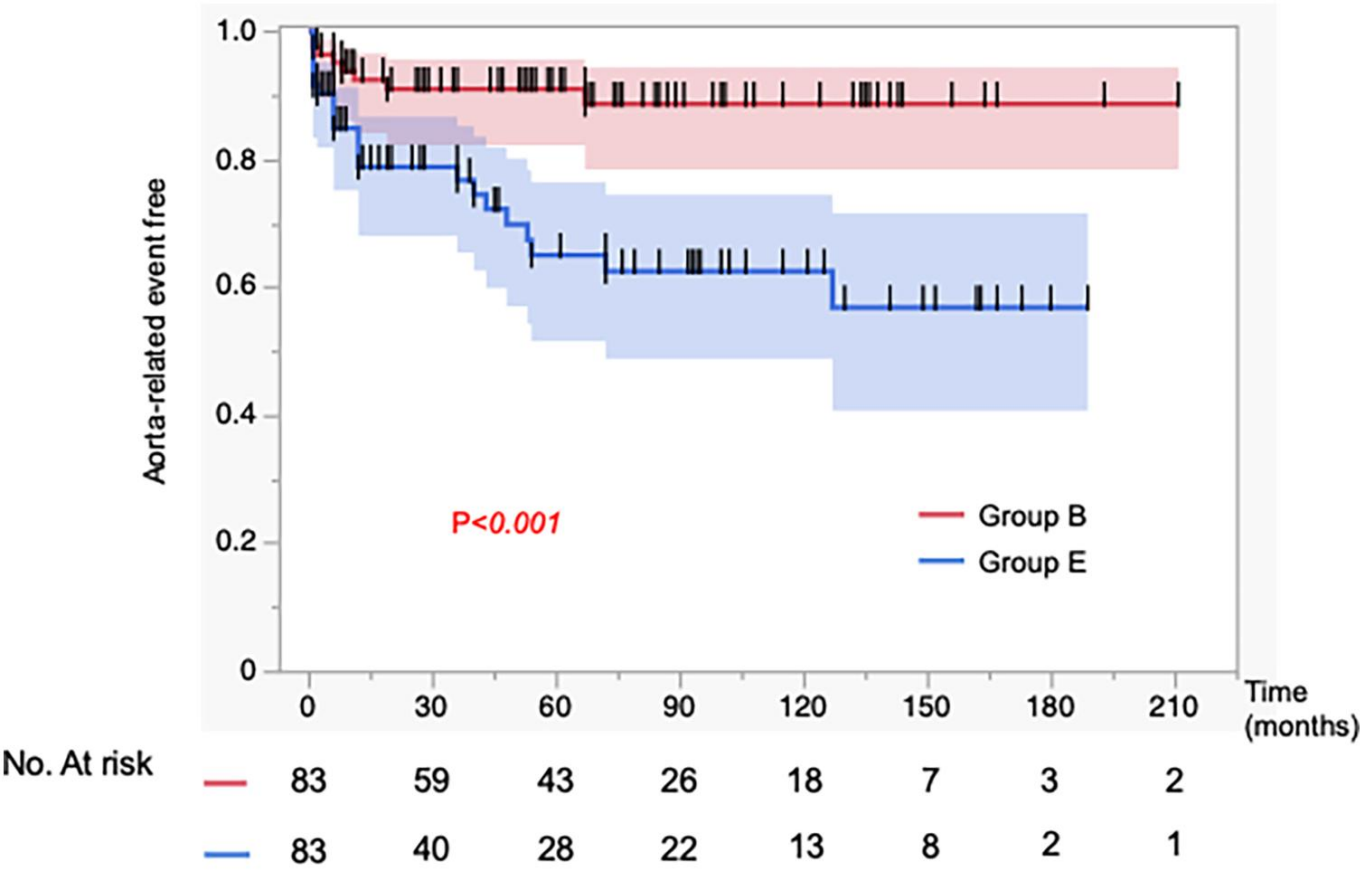
Kaplan–Meier curves for the incidence of aorta-related events in 83 matched patients. Propensity score matching was performed to adjust for baseline characteristics. The incidence of aorta-related events was significantly higher in the aortic expansion group (Group E) compared to the best medical therapy group (Group B).

**Figure 3:**
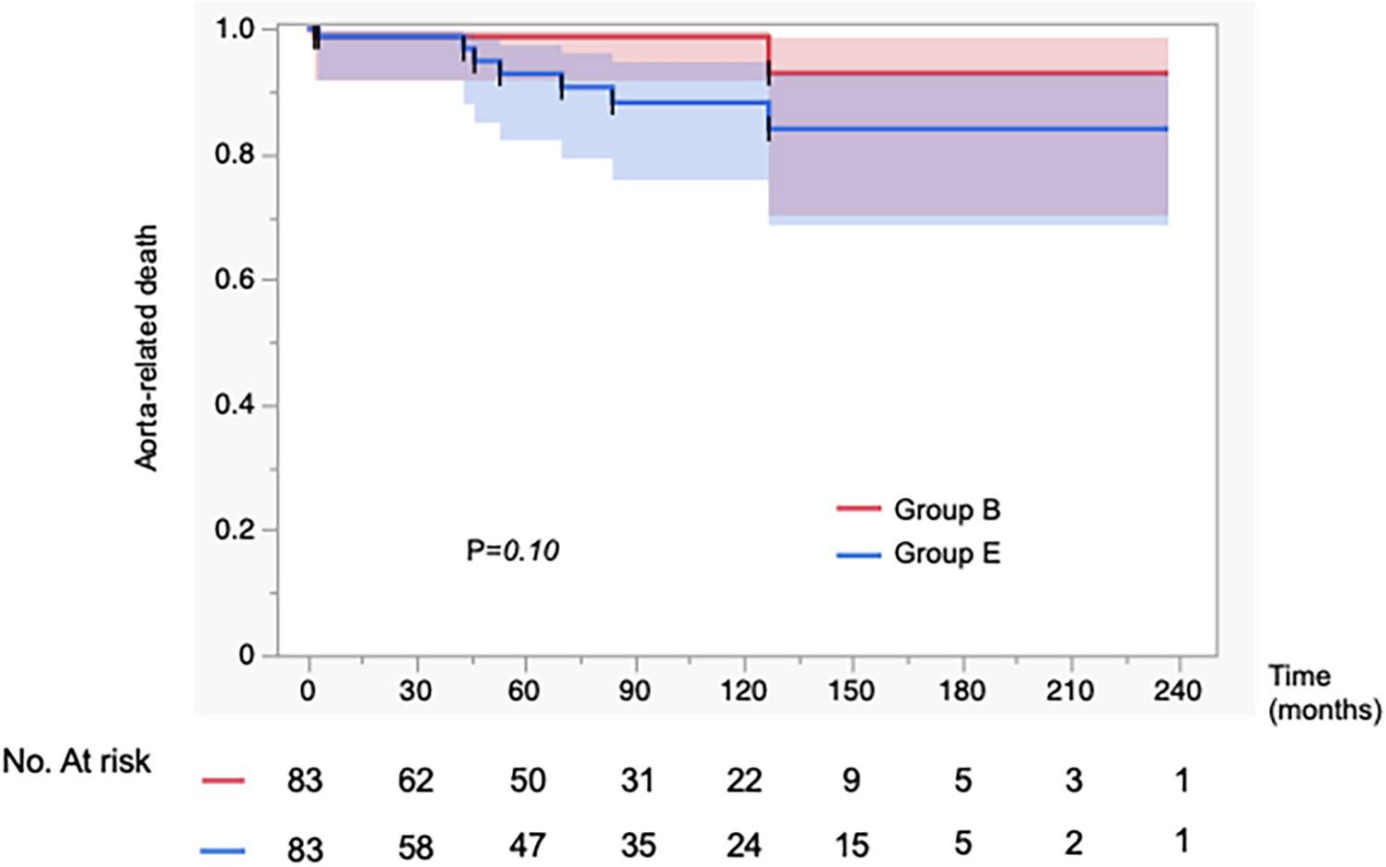
Kaplan–Meier curves showing the incidence of aorta-related mortality in 83 propensity score–matched patients. Propensity score matching was performed to adjust for baseline characteristics. There was no significant difference in the incidence of aorta-related mortality between the aortic expansion group (Group E) and the best medical therapy group (Group B).

In Figure 2, the incidence of aorta-related events was higher in Group E than in Group B, showing a significant difference between the two groups (*p* = 0.0006). In contrast, as shown in Figure 3, the aorta-related death–free rates (Group B vs. Group E at 1/5/10 years: 99/99/99% vs. 99/93/88%) demonstrated only a trend toward poorer outcomes in Group E, without reaching statistical significance (*p* = 0.097). In Group E, seven aortic-related deaths were identified. One patient who underwent TEVAR for aneurysmal degeneration in the chronic phase subsequently developed retrograde type A dissection and died of cardiac tamponade. Another patient died following thoracoabdominal aortic repair performed for chronic dissecting aneurysm. The remaining patients died of ruptured aortic aneurysms, for which invasive treatment was not pursued due to clinical or personal constraints (Supplementary Table 3). These events underscore the persistent challenges of managing chronic-phase aneurysmal progression and the limitations of available therapeutic strategies.

## Discussion

From our investigation, we obtained the following novel insights: (1) In patients with subacute to early chronic Stanford type B aortic dissection and an aortic diameter expansion of ≤5 mm over 6 months, preemptive TEVAR showed favorable outcomes when anatomical criteria were met, with acceptable long-term survival rates. (2) In cases with slow aortic enlargement, a combined approach of TEVAR and BMT was associated with good long-term prognosis. (3) In cases with slow aortic enlargement, aneurysmal dilatation continued to progress during long-term follow-up, and the extent of aortic expansion was significantly larger compared to the BMT group.

With advances in endovascular therapy, the classification of subacute type B aortic dissection has become increasingly refined. It is now broadly divided into two main categories: the “complicated type,” which presents with vascular-related complications, and the “uncomplicated type,” characterized by relative clinical stability without life-threatening features (12–14). Among the uncomplicated cases, a subset is further identified as “high-risk uncomplicated type,” in which future expansion of the false lumen is anticipated based on anatomical or morphological indicators (11, 15, 16). However, a major concern is that preemptive TEVAR, when performed to prevent future aneurysmal degeneration, cannot be universally applied to all cases of type B aortic dissection with patent false lumen. Currently, there is no established evidence or consensus regarding the criteria for selecting appropriate candidates for this preventive intervention (1). Several predictors of aortic enlargement have been reported, including the number of vessels arising from the false lumen, number of intercostal arteries, entry tear on the lesser curvature, false lumen diameter >22 mm, large entry tear (≥10 mm), and narrow true lumen (11, 12, 17–19). Among these, large aortic diameter (≥40 mm) is of particular clinical relevance, as morphological changes related to aortic diameter are directly associated with aneurysmal progression and can serve as a key indication for intervention (12, 20). Although current guidelines define an aortic diameter increase exceeding 5 mm over 6 months as an absolute indication for intervention (1, 7), preemptive TEVAR is generally recommended during the subacute phase (10). This discrepancy contributes to variability in clinical decision-making across institutions and among individual operators. In this study, we evaluated clinical outcomes in patients with type B aortic dissection who exhibited aortic enlargement within 6 months of onset but did not meet the ≥5 mm/6 months threshold for rapid expansion. These patients were managed either with preemptive TEVAR or continued BMT, allowing us to assess the potential benefits and limitations of early intervention in cases where morphological progression is evident, yet conventional criteria for intervention are not strictly met. In patients with progressive aortic enlargement who remained on BMT, progression to aneurysmal degeneration was observed. Importantly, the clinical relevance of aortic enlargement as a prognostic marker is supported by our previous findings, in which a maximum aortic diameter greater than 40 mm was associated with a significantly increased risk of late aortic adverse events in patients with type B dissection (12). This diameter threshold may therefore serve as a practical morphological indicator of heightened risk and could help identify patients who may benefit from consideration of preemptive intervention before irreversible aneurysmal degeneration occurs. In contrast, those who underwent TEVAR based on favorable anatomical criteria experienced a low incidence of subsequent complications and demonstrated favorable false lumen thrombosis. These findings suggest that even in cases with slow aortic expansion, preemptive TEVAR may be beneficial in selected patients. Our results support the therapeutic potential of early endovascular intervention and reinforce the value of individualized assessment in guiding treatment decisions for subacute type B aortic dissection. Our study has several limitations, including its retrospective design, relatively small sample size, incomplete follow-up (6.2%), and variability in the number of CT scans performed among patients. The decision to perform preemptive TEVAR was left to the discretion of the attending surgeons, potentially introducing selection bias in the comparison between BMT and intervention groups. Although we attempted to minimize this bias through propensity score matching—including demographic and vascular risk factors such as body size, sex, hypertension, and diabetes—residual confounding cannot be completely excluded. In addition, preemptive TEVAR includes a variety of approaches, such as stent graft placement in Zone 2, Zone 3, and more distal portions of the descending thoracic aorta, each of which may carry different levels of risk. Future investigations should therefore consider stratifying cases by anatomical zone and intervention strategy to enable more risk-adjusted evaluation. Another limitation of this study is that we did not perform a stratified analysis according to the specific procedural approaches of Group E, which may have obscured differences in efficacy and procedural risk among these treatment plans. This study should be regarded as hypothesis-generating, and prospective validation in larger cohorts is warranted. Although all interventions were performed in accordance with the instructions for use of the stent grafts, further studies are needed to identify which patient subgroups may truly benefit from preemptive TEVAR in terms of long-term outcomes.

## Conclusions

Selective preemptive TEVAR in patients with rapid aortic enlargement during the subacute to early chronic phases of type B aortic dissection showed favorable outcomes, including false lumen thrombosis. Although aorta-related mortality was not significantly different from the group of best medical therapy, the increased risk of rupture in untreated patients suggests a potential benefit. Further prospective studies are warranted to refine patient selection criteria and determine the optimal timing for intervention.

## Data Availability

The original contributions presented in the study are included in the article/Supplementary Material, further inquiries can be directed to the corresponding author.
